# Correlation of Common Carotid Artery Blood Flow Parameters With Transthoracic Echocardiographic Cardiac Output for Assessing Fluid Responsiveness After Passive Leg Raising (PLR) Test in Critically Ill Patients

**DOI:** 10.7759/cureus.40229

**Published:** 2023-06-10

**Authors:** Rohit Patnaik, Bhuvana Krishna, Sriram Sampath

**Affiliations:** 1 Critical Care Medicine, Institute of Medical Sciences and SUM Hospital, Bhubaneswar, IND; 2 Critical Care Medicine, St. John's Medical College and Hospital, Bengaluru, IND

**Keywords:** fluid responsiveness, transthoracic echocardiography, lvot vti, passive leg raising test, carotid doppler

## Abstract

Introduction

The passive leg raising (PLR) test is a simple, non-invasive method of knowing fluid responsiveness by acting as an internal-fluid challenge. The PLR test coupled with a non-invasive assessment of stroke volume would be the ideal method to assess fluid responsiveness. This study aimed to determine the correlation between transthoracic echocardiographic cardiac output (TTE-CO) and common carotid artery blood flow (CCABF) parameters in determining fluid responsiveness with the PLR test.

Methods

We performed a prospective observational study on 40 critically ill patients. Patients were evaluated with a 7-13 MHz linear transducer probe for CCABF parameters calculated using time-averaged mean velocity (TAmean) and with a 1-5 MHz cardiac probe equipped with tissue doppler imaging (TDI) for TTE-CO calculated using left ventricular outflow tract velocity time integral (LVOT VTI) with an apical five-chamber view. Two separate PLR tests (five minutes apart) were done within 48 hours of ICU admission. The first PLR test was to assess the effects on TTE-CO. The second PLR test was performed to assess the effects on CCABF parameters. Patients were designated as fluid responders (FR) if changes in TTE-CO (Δ TTE-CO) ≥ 10 %.

Results

A positive PLR test was observed in 33% of patients. A strong correlation was present between absolute values of TTE-CO calculated using LVOT VTI and the absolute values of CCABF calculated using TAmean (r=0.60, p<0.05). However, a weak correlation was found between Δ TTE-CO and changes in CCABF (Δ CCABF) during the PLR test (r=0.05, p<0.74). A positive PLR test response could not be detected by Δ CCABF (area under the curve (AUC): 0.59 ± 0.09).

Conclusions

We found a moderate correlation between TTE-CO and CCABF at baseline. However, Δ TTE-CO had a very poor correlation with Δ CCABF, during the PLR test. Considering this, CCABF parameters may not be recommended as a means to detect fluid responsiveness with PLR tests in critically ill patients.

## Introduction

When it comes to treating patients who are in shock, fluid resuscitation has proved crucial. Organ dysfunction and tissue hypoperfusion may result from insufficient fluid intake. Fluid overload may lengthen the time spent in the intensive care unit and prolong the need for mechanical ventilation [1]. To administer the right amount of fluids, we need to know if the patient is fluid responsive i.e., increases stroke volume/cardiac output on giving a fluid challenge.

Dynamic assessments of fluid responsiveness are advised to identify which patients are likely to increase stroke volume with the administration of fluids. This rule holds true for all patients and diseases. The passive leg raising (PLR) test, one of the many dynamic measurements of fluid responsiveness, is a straightforward, non-invasive technique for determining fluid responsiveness by serving as an internal-fluid challenge. The primary benefit of the PLR technique is that it is entirely reversible [2,3].

Over the past 20 years, a variety of dynamic methods based on heart-lung interactions have been developed to predict fluid responsiveness. It is true that assessing the central venous pressure or pulse pressure variation is not feasible in many critically ill patients, but each of the following tools such as transthoracic echocardiogram (TTE), lung ultrasound, and arterial blood pressure monitoring provide important information about fluid status, even if the patient is not receiving mechanical ventilation. [4].

Several non-invasive/minimally invasive methods have been developed to assess stroke volume and cardiac output [4-7]. Bedside sonography is one such simple, non-invasive method.

The PLR test would be the best approach to evaluate fluid responsiveness in critically ill patients and direct our fluid administration, along with a non-invasive assessment of stroke volume/cardiac output. The left ventricular outflow tract (LVOT) velocity time integral (VTI) change with a PLR test has been found to be specific in predicting fluid responsiveness [8,9]. However, getting an acceptable window of the LVOT and an appropriate apical five-chamber view can be difficult, especially in emergencies or when adequate views are impossible because of body habits, incisions/wound dressings, or other obstructions [10,11].

Doppler measurement of blood flow in the carotid artery may estimate changes in cardiac output during a PLR test [12,13]. Correlation between common carotid artery blood flow (CCABF) Doppler parameters with TTE cardiac output (CO) using LVOT VTI to assess the effects of the PLR test in predicting fluid responsiveness has not been demonstrated in any group of patients. When evaluating fluid responsiveness with the PLR test, CCABF parameters could provide a significantly simpler substitute for TTE-CO. Our study aimed to try and fill in these lacunae. The primary objective of our study was to determine the correlation between CCABF parameters and TTE-CO in determining volume responsiveness with the PLR test. The secondary objective of our study was to determine whether changes in CCABF (Δ CCABF) parameters can detect a positive PLR test.

## Materials and methods

This study was a single-center prospective observational study conducted in the medical ICU of a tertiary care hospital, St. John's Medical College Hospital, Bengaluru, India. It was initiated after taking approval from the Institutional Ethics Committee of St. John's Medical College and Hospital (approval number: 3/2019) and registration with the Clinical Trials Registry of India (CTRI/2019/04/018828). The study duration was 18 months. The study was conducted according to the STROBE (STrengthening the Reporting of OBservational studies in Epidemiology) guidelines and checklist.

Patients above 18 years of age who were spontaneously or mechanically ventilated were included in the study. Exclusion criteria consisted of age less than 18 years, those with cardiac arrhythmias at the time of measurement, carotid artery stenosis >50 % or occluded carotid arteries, any contraindications to PLR (head injury or intracranial hypertension, lower limb amputations, on non-invasive positive pressure ventilation, wearing venous compression stockings, intraabdominal hypertension, a poor ultrasonographic window for carotid Doppler measurements, and refusal of consent.

**Figure 1:**
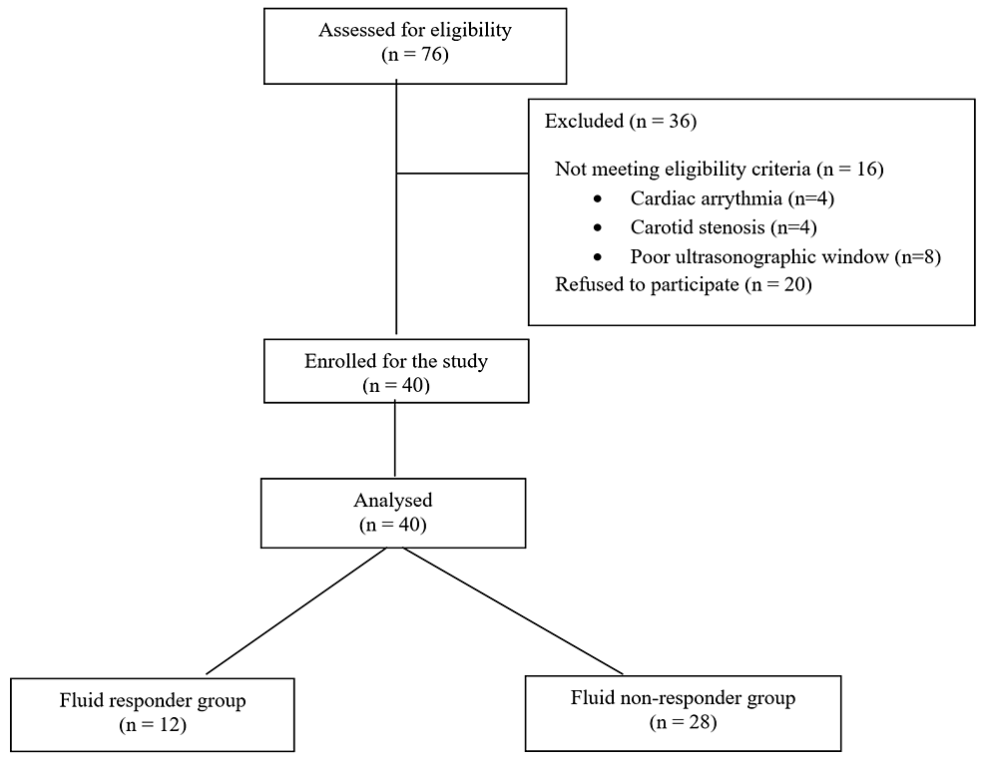
Flow diagram of patients

Patients admitted to the medical ICU were screened within 48 hours of admission, and those who met inclusion and exclusion criteria were included in the study (Figure 1). Written informed consent was taken from the patient or the patient’s surrogate decision maker. Demographic details, admission diagnosis, Acute Physiology and Chronic Health Evaluation II (APACHE II) score, Sequential Organ Failure Assessment (SOFA) score, and comorbidities score were recorded. All ultrasound-based evaluations were performed by the principal investigator, who was trained in basic critical care echocardiography. Patients were evaluated with a 7-13 MHz linear transducer probe for CCABF parameters and with a 1-5 MHz phased array probe equipped with tissue Doppler imaging (TDI) for TTE-CO using LVOT VTI with an apical five-chamber view. We used the Sonosite EDGE® ultrasound machine (FUJIFILM Sonosite, Inc., Bothell, Washington, United States) for all our measurements.

**Figure 2:**
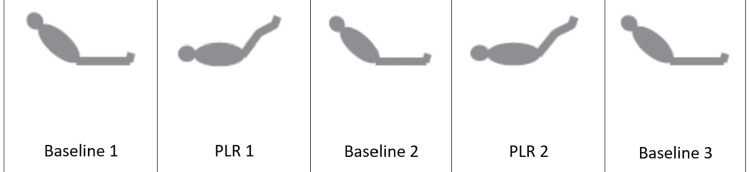
Position of the patient during the two PLR maneuvers PLR, passive leg raising

We measured hemodynamic and ultrasound data during five sequential steps (Figure 2, Table 1). A first set of measurements were obtained in the semi-recumbent position (45°) (designated Baseline 1). Using an automatic bed elevation technique, the lower limbs were then raised to a 45° angle while the patient’s trunk was lowered in the supine position. Thus, the angle between the trunk and the lower limbs remained unchanged (135°). A second set of measurements (designated PLR 1) was obtained during leg elevation within one minute of performing PLR 1. The body posture was then returned to the baseline 1 position and a third set of measurements were recorded (designated Baseline 2). After five minutes of returning to Baseline 2, a second PLR maneuver was performed similar to PLR 1 described above. A fourth set of measurements were recorded at this time within one minute of performing PLR 2 (designated PLR 2). The body posture was then returned to the Baseline 2 position and a fifth final set of measurements was recorded (Baseline 3). The ventilator settings and vasoactive therapy were kept constant throughout the five sets of recordings in the study period. No new fluid administration was done and all ongoing fluid therapy was stopped during the conduct of the five sets of recordings. A second intensivist who was not part of the study recorded all the parameters. During PLR 1, TTE-CO parameters were recorded and in PLR 2, CCABF parameters were recorded.

**Table 1:** Method of the two PLR maneuvers with measurements LVOT, left ventricular outflow tract; LVOT VTI, left ventricular outflow tract velocity-time integral; TAmean, time-averaged mean velocity; TApeak, time-averaged peak velocity; PSV; peak systolic velocity †, Indicates parameter was measured ‡, Indicates parameter was not measured

Parameter	Baseline 1	PLR 1	Baseline 2	PLR 2	Baseline 3
LVOT Diameter	†	‡	‡	‡	‡
LVOT VTI	†	†	†	‡	†
TAmean (Right)	†	‡	†	†	†
TApeak (Right)	†	‡	†	†	†
Carotid Diameter (Right)	†	‡	†	†	†
PSV (Right)	†	‡	†	†	†
TAmean (Left)	†	‡	†	†	†
TApeak (Left)	†	‡	†	†	†
Carotid Diameter (Left)	†	‡	†	†	†
PSV (Left)	†	‡	†	†	†

**Figure 3:**
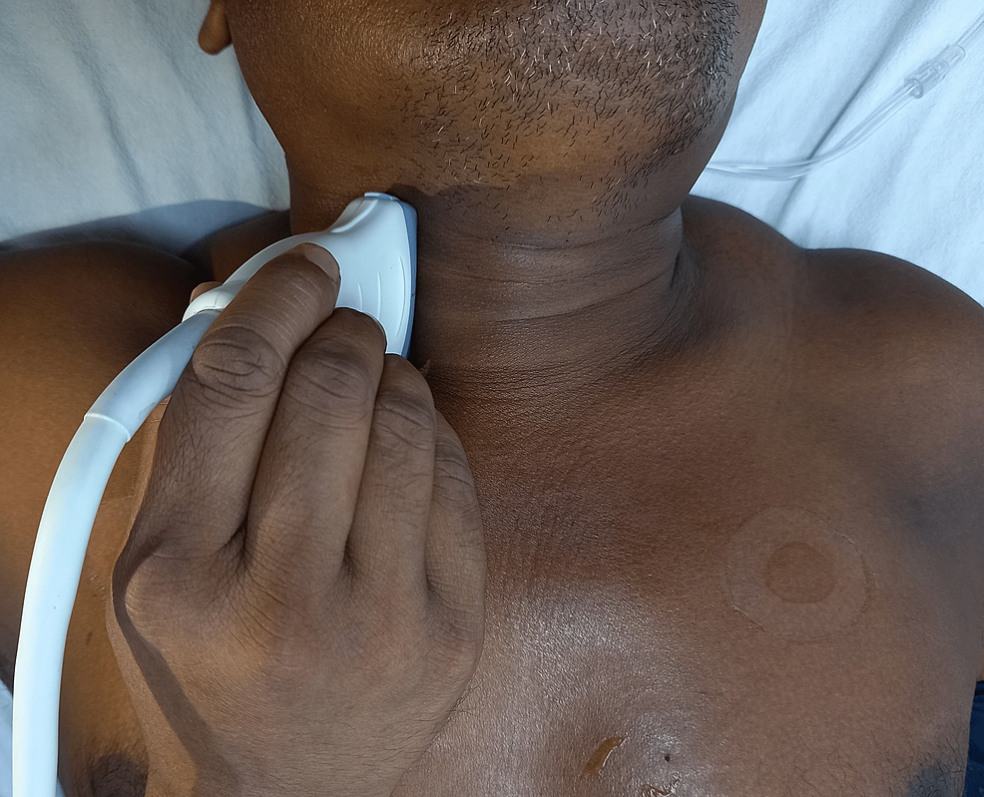
Probe position for carotid Doppler measurements (anterior view)

**Figure 4:**
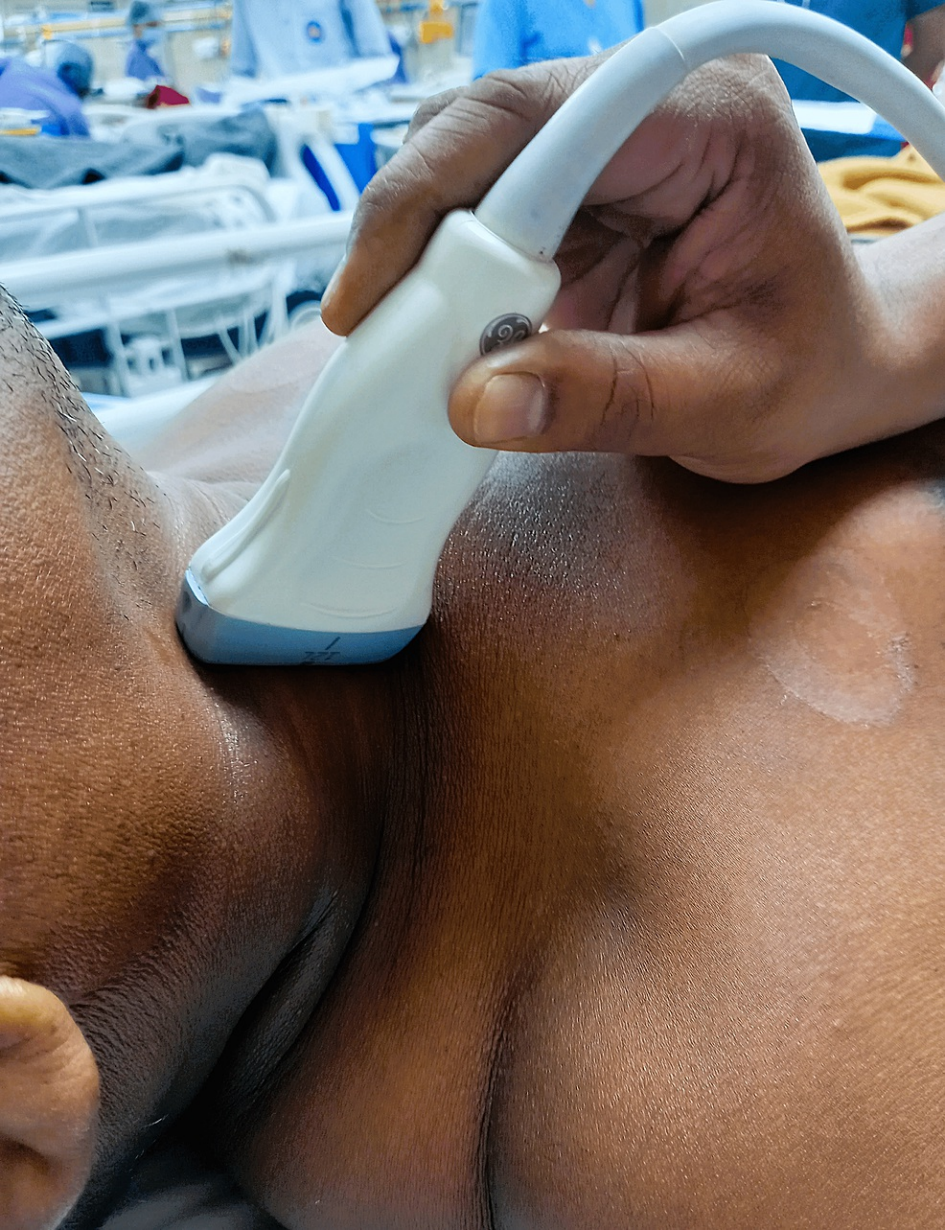
Probe position for carotid Doppler measurements (lateral view)

**Figure 5:**
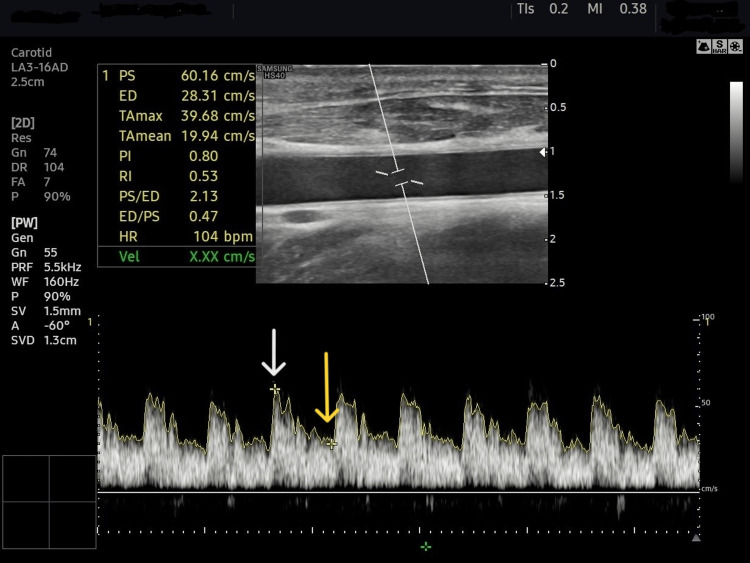
Schematic presentation of Doppler findings of common carotid artery. PS, peak systolic velocity (white arrow); ED, end-diastolic velocity (yellow arrow); TAmean, time-averaged mean velocity; TAmax, time-averaged peak velocity; PI, pulsatility index; RI, resistivity index; HR, heart rate

Doppler measurements of both common carotid arteries were obtained at the level of the thyroid gland with the head rotated away from the ultrasound operator (Figures 3, 4). Using pulsed wave Doppler, the sampling volume was positioned in the middle of the lumen, with a 1 mm caliper, placed parallel to the vessel walls in the center of the laminar flow identified by color flow ultrasound (Figure 5) [12,14]. Each of the CCABF parameters was taken as the mean of five consecutive beats of a complete respiratory cycle.

**Figure 6:**
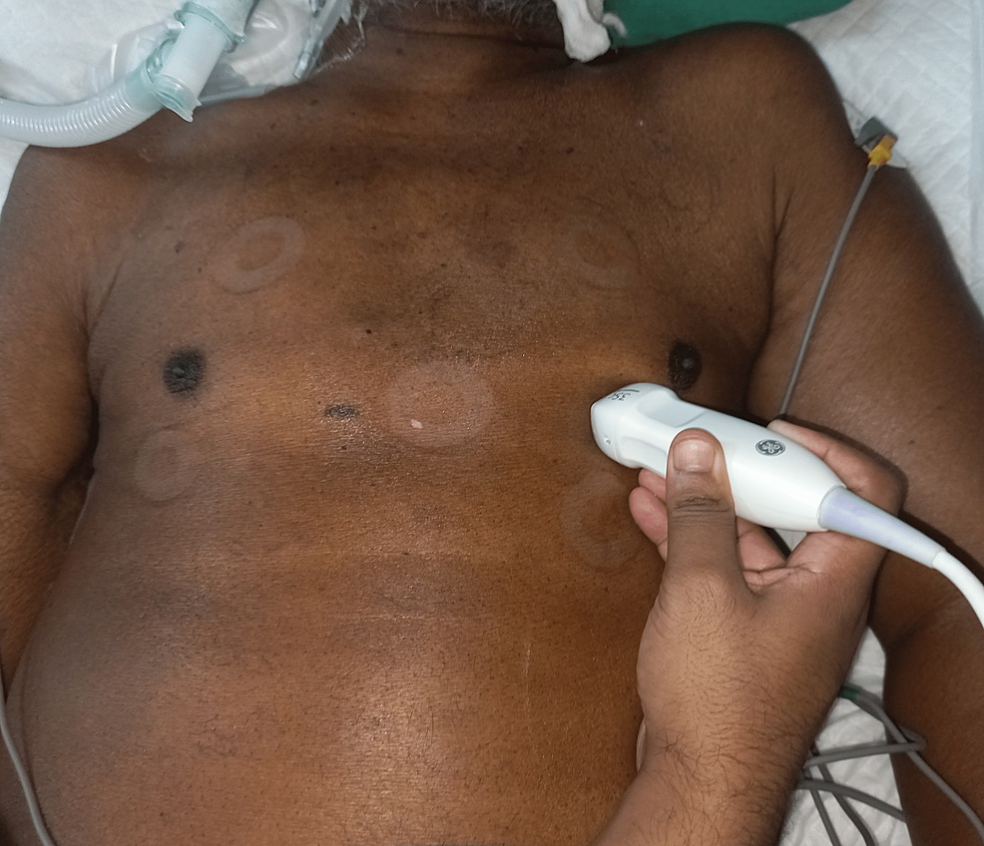
Probe position for apical five-chamber view (anterior view)

**Figure 7:**
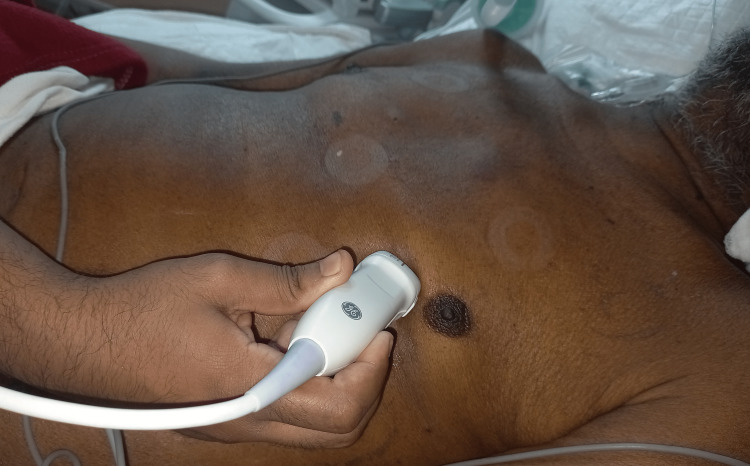
Probe position for apical five-chamber view (lateral view)

**Figure 8:**
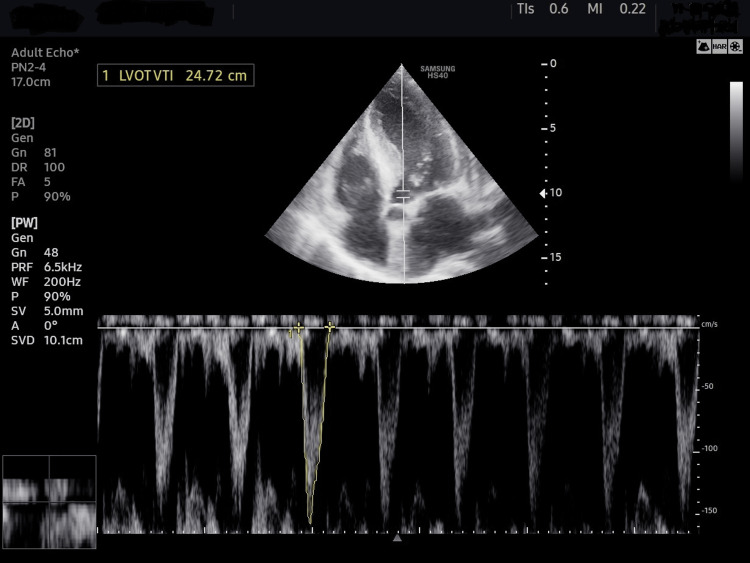
Schematic presentation of LVOT VTI measurement in apical five-chamber view (area under yellow tracing) LVOT VTI, left ventricular outflow tract velocity time integral

LVOT diameter was measured once at Baseline 1, 0.5 cm below the level of the aortic valve at the point of maximum separation in the parasternal long axis view (PLAX) view. TTE-CO using LVOT VTI was measured in apical five-chamber view (A5C) 0.5 cm below the level of the aortic valve (Figures 6-8). The LVOT VTI was determined from the mean of five consecutive beats of a complete respiratory cycle. Patients were designated as fluid responders if the change in TTE-CO (Δ TTE-CO) ≥ 10 %. The calculations used have been shown in Table 2 [12].

**Table 2:** Calculations A, area of left ventricular outflow tract; r, radius of left ventricular outflow tract; SV, stroke volume; HR, heart rate; TTE-CO, transthoracic echocardiography based cardiac output; LVOT VTI, left ventricular outflow tract velocity-time integral; Δ LVOT VTI, change in LVOT VTI; Δ TTE-CO, change in TTE-CO; CCABF volume, common carotid artery blood flow volume; Δ CCABF volume, change in common carotid artery blood flow volume; D, diameter of CCA; TAmean, time-averaged mean velocity; TApeak, time-averaged peak velocity; Δ TAmean, change in TAmean; Δ TApeak, change in TApeak † Patients were designated as Fluid Responders (FR) if Δ TTE-CO ≥ 10 % [8]

Parameter (Units)	Formula
A (cm^2^)	π x r^2^
SV (ml)	A (cm2) x LVOT VTI (cm)
TTE-CO (mL/min)	SV x HR
Δ LVOT VTI (cm)	(LVOT VTI PLR – LVOT VTI Baseline) / LVOT VTI Baseline
Δ TTE-CO (mL/min)	(TTE-CO PLR – TTE-CO Baseline) / TTE-CO Baseline
CCABF (mL/min)	D (cm) x TAmean
Δ CCABF (mL/min)	(CCABF PLR – CCABF Baseline) / CCABF Baseline
Δ TAmean	(TAmean PLR – TAmean Baseline) / TAmean Baseline
Δ TApeak	(TApeak PLR – TApeak Baseline) / TApeak Baseline

Statistical analysis

Considering data from previous studies, taking Pearson’s correlation coefficient r=0.54 between absolute values of TTE-CO and absolute values of CCABF calculated from TAmean, a sample size of 40 was calculated [15,16]. Good intraobserver and interobserver repeatability has been demonstrated in previous studies (interobserver coefficient of variation of 4.8% and intraobserver coefficient of variation of 3.1%) [17].

Data were expressed as mean±SD, median and interquartile range (IQR) or as number and percent frequency, as appropriate. All data were recorded and analyzed using Stata Statistical Software Release 13 (StataCorp LP., College Station, Texas, United States). A p-value below 0.05 was chosen for statistical significance. Student’s t-test was used for continuous variables, chi-square test was used for categorical variables, Pearson’s correlation coefficient was used for the correlation between CCABF parameters and TTE-CO parameters. Multivariate logistic regression analysis was used to evaluate the ability of CCABF parameters to predict PLR positivity. Shapiro-Wilk test of normality was used to determine the normality of distribution. Quantitative variables were presented as mean±SD plus 95%CI and categorical variables as frequency (percentage). A multivariate logistic regression model was done to assess the variables positively associated with TTE-CO.

## Results

Fifty-two patients were screened over a period of 20 months and 40 patients were recruited. Of the 40 patients, 28 were male and 12 were female (Table 3). Although our ICU has both medical and surgical patients, all of the 40 patients were medical. The Δ TTE-CO using LVOT VTI was used to classify them as fluid responders or non-responders. There were no patients who were excluded due to their inability to perform the PLR tests.

**Table 3:** Baseline parameters All values are expressed as mean ± SD unless otherwise noted SOFA, Sequential organ failure assessment; IQR, Interquartile range

Parameter	Fluid Responders n = 12	Fluid Non-Responders n = 28	P-value
Age (Years)	47.00 ± 16.12	46.93 ± 16.44	0.62
Sex (M:F)	6:6	22:6	0.13
SOFA	9.17 ± 4.60	11.11 ± 3.94	0.13
Length of ICU stay (Days)	9.08 ± 5.56	9.79 ± 5.98	0.85
Mechanical Ventilation (%)	10 (92%)	25 (90%)	0.62

Demographic parameters were comparable between fluid responders and fluid non-responders (Table 3). The need for vasopressors was higher whereas cardiac output was lower in the fluid responders (Table 4). CCA blood flow based on TAmean and TA peak was lower in the fluid responders (Table 5).

**Table 4:** Baseline hemodynamic parameters All values are expressed as mean±SD unless otherwise noted SBP, systolic blood pressure; DBP, diastolic blood pressure; A, area of left ventricular outflow tract; LVOT VTI, left ventricle outflow tract velocity time integral; TTE-CO, transthoracic echocardiography based cardiac output; SD, standard deviation

Parameter	Fluid Responders n=12	Fluid Non-Responders n=28	P-value
Vasopressor (%)	5 (41%)	7 (25%)	0.45
SBP (mm Hg)	111.08 ± 18.38	127.21 ± 23.81	0.09
DBP (mm Hg)	64.58 ± 13.76	69.92 ± 12.78	0.33
A (cm^2^)	2.42 ± 0.46	2.87 ± 0.54	0.32
LVOT VTI (cm)	17.00 ± 5.00	18.57 ± 4.73	0.30
TTE-CO (ml/min)	4040.72 ± 1526.80	5314.37 ± 2262.00	0.07

**Table 5:** Baseline CCABF parameters All values are expressed as median (IQR) unless otherwise noted D, diameter of common carotid artery; TA mean, time-averaged mean velocity; TA peak, time-averaged peak velocity; CCABF, common carotid artery blood flow; IQR, interquartile range

Parameter	Fluid Responders	Fluid Non-Responders	P-value
D (cm)	0.61 (0.54 - 0.67)	0.61 (0.55 - 0.64)	0.10
TAmean (cm/sec)	14.7 (12.92 - 17.15)	22.25 (16.05 - 28.2)	0.07
TApeak (cm/sec)	23.9 (22.12 - 35.87)	40.45 (23.07 - 49.25)	0.12
CCABF using TAmean (ml/min)	295.72 (232.20 - 355.80)	323 (246.46 - 490.48)	0.34
CCABF using TApeak (ml/min)	475.61 (356.84 - 651.71)	570 (379.81 - 811.64)	0.24

**Figure 9:**
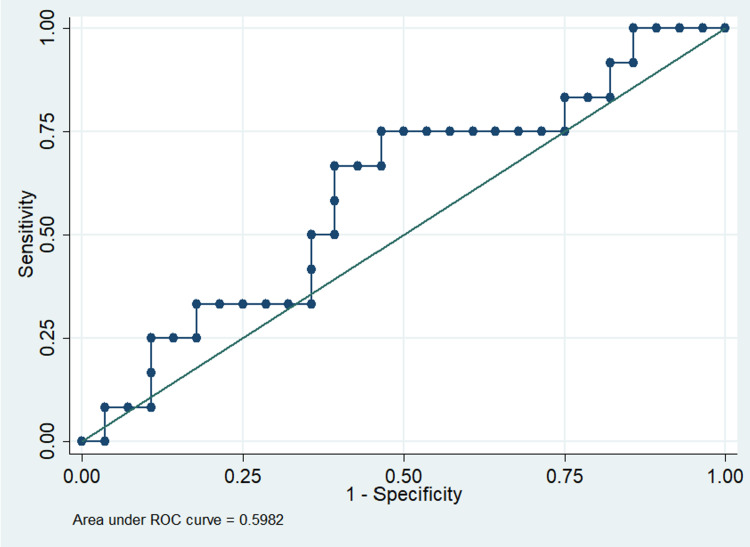
ROC curve for predicting fluid responsiveness with change in CCA blood flow CCA, common carotid artery; ROC, receiver operating characteristic

Moderate positive correlation was present between absolute values of TTE-CO calculated using LVOT VTI and absolute values of CCABF using TAmean (r=0.60, p<0.05) (Table 6). However, poor positive correlation was present between Δ TTE-CO calculated using LVOT VTI with PLR test and Δ CCABF using TAmean with PLR test (r=0.60, p<0.05) (Table 7). The receiver operating characteristic (ROC) curve for predicting fluid responsiveness with Δ CCABF showed a poor area under the ROC curve (AUROC) of 0.59 (Figure 9). A multivariate logistic regression analysis was run to predict TTE-CO at baseline using CCABF, CCA peak systolic velocity, systolic blood pressure, and heart rate (Table 8). Out of the four variables, only two (CCABF and heart rate) added statistically significantly to the prediction of TTE-CO (F (4, 35) = 8.19, p < 0.0005, R2 = 0.48). Regression analysis was also done to predict Δ TTE-CO with the PLR test, none added statistically significantly to the prediction.

**Table 6:** Correlation of TTE-CO with CCABF CCABF, common carotid artery blood flow; TTE, transthoracic echocardiography; CO, cardiac output

	CCABF	
	Pearson’s Correlation coefficient	P-value
TTE-CO	r = 0.60	0.05

**Table 7:** Correlation of change in TTE-CO with change in CCABF CCABF, common carotid artery blood flow; TTE, transthoracic echocardiography; CO, cardiac output

	Changes in CCABF	
	Pearson’s Correlation coefficient	P-value
Changes in TTE-CO	r = 0.05	0.74

**Table 8:** Application of multivariate logistic regression analysis in determining factors predicting cardiac output at baseline CCABF, common carotid artery blood flow; CCA, common carotid artery; TAmean: time-averaged mean velocity

	Regression Coefficient (95 % Confidence Interval)	Standard Error	P-value
CCABF (TAmean)	3.44 (1.13 – 5.75)	1.13	0.005
CCA peak systolic velocity	3.00 (-15.82 – 21.82)	9.27	0.748
Systolic blood pressure	17.19 (-6.09 – 40.48)	11.47	0.143
Heart rate	25.96 (2.98 – 48.95)	11.32	0.028

## Discussion

This was a single-center, observational study done to assess the correlation of CCABF parameters with TTE parameters in a mixed ICU. 33% of the patients were fluid responsive. There was no significant difference between fluid responders and non-responders with respect to age, sex, or SOFA scores.

The hemodynamic variables between fluid responders and non-responders were compared. Between the two groups, with respect to baseline hemodynamic data, fluid responders had more vasopressor support, lesser cardiac output, and lesser CCABF. However, there was no difference in mean mean arterial pressure (MAP) between the two groups. 

Previous studies have noted fluid responsiveness in approximately 50% of critically ill patients [18]. The difference in baseline hemodynamic variables has also been noted in other studies [19]. So our study was in agreement with previous studies of such nature.

Our study showed a moderate positive correlation was present between absolute values of TTE-CO calculated using LVOT VTI and absolute values of CCABF using TAmean, at baseline. However, a poor positive correlation was present between Δ TTE-CO and Δ CCABF. Although our study showed the poor ability of the CCABF measurements to track Δ TTE-CO, this could have been plausible due to several reasons. Firstly, the measurement of cardiac output is most reliable when obtained at end-expiration [20]. However, considering that both TTE-CO and CCABF measurements were single-time point measurements, it was impossible to time the measurements with end-expiration. To circumvent this, we took an average of three measurements for both the TTE-CO and CCABF measurements in all our patients. Secondly, although we took all technical considerations as mentioned by Blanco into consideration, Doppler measurements may not be reliable with higher blood flow velocities where flow-mediated vasodilatation (FMD) might occur [21,22]. In the current study, more than 80% of the patients had higher blood flow velocities (> 400 ml/min) [23]. Although we did not find any significant change in carotid diameter with the PLR test, the higher blood flow velocities may have been one of the reasons for the poor correlation in CCABF parameters to track changes in CO. Thirdly, as mentioned in previous studies, the proportion of CO that is directed towards the carotid artery may vary depending on cerebral blood flow (CBF) regulation. CBF is tightly regulated with selective redistribution to the brain during low perfusion states. This suggests that probably Δ CCABF might be independent of changes in CO. Rather, it might solely be dependent on autoregulation to maintain CBF. Fourthly, atherosclerosis and narrowing of the vessels might be an additional confounder. However, to remove this bias, we excluded patients with carotid artery stenosis >50 % and occluded carotid arteries/visible carotid plaque. Fifthly, it has been noted in the literature that significant intra-observer variability might be present when performing CCABF measurements [24]. We had done a pilot study which revealed an intra-observer variability of 4.8%. Thus, the contribution of this is at best marginal.

We found moderate positive correlation between TTE-CO and CCABF at baseline. Similar to our study findings, Peng et al. compared CCABF-derived CO and TTE-CO, which showed moderate agreement, although they did not perform any PLR test to know whether any Δ TTE-CO could be tracked accurately by Δ CCABF [25]. To circumvent this, Girotto et al. performed CCABF measurements and compared them with invasive CO measurements rather than TTE-CO [16]. Although they found carotid blood flow measurements to be unreliable, they compared a non-invasive and invasive method. Whereas, we compared two non-invasive methods. Our study showed a poor positive correlation between Δ TTE-CO and CCABF with the PLR test. In contrast to our study, Marik et al. found a strong correlation between change in CO assessed by TTE with Δ CCABF parameters, particularly in fluid responders [26]. However, the increase in CCABF with the PLR test could have been attributed to the flow-mediated dilatation (FMD) that the authors have described. In our study, we did not find any significant change in carotid diameter with the PLR test. This FMD has been regarded as a marker of endothelial integrity. Our study had a much higher mean SOFA score compared to Marik et al.' study. Higher SOFA scores indicate sicker patients and consequent lesser endothelial integrity. This could have been the reason why we did not observe any FMD in our study. The strength of our study was that we followed the technique of recording CCABF parameters meticulously. Several valid concerns about the technique have been raised by Blanco, but we incorporated most of the technical considerations while doing volumetric CCABF measurements [14].

CCABF measurements at baseline have been incorporated into shock assessment protocols in the ER. The Rapid Ultrasound in Shock (RUSH)-velocity time integral protocol has been one of them [22]. Although it has not been incorporated into widespread use, our study shows that CCABF measurements at baseline can be used to assess the severity of shock. This stems from the fact that there was a moderate positive correlation between TTE-CO and CCABF at baseline. Since the carotid artery is a very superficial structure, it would be much less time-consuming to assess it in time-constrained settings such as the ER. However, caution must be exercised when using CCABF measurements in determining fluid responsiveness considering that Δ CCABF have poor ability to track changes in TTE-CO. 

Our study had a few limitations. Firstly, we measured the Δ TTE-CO and the Δ CCABF parameters by incorporating two different PLR tests. Thus, both the measurements of TTE-CO and CCABF parameters were not done simultaneously. We treated the PLR tests as being equivalent to each other. Better comparisons could be made if both measurements were to be done simultaneously in a single PLR test. However, this was not feasible in this study due to logistical reasons. Secondly, as previously mentioned, the measurement of TTE-CO and CCABF could not be timed with end-expiration. Considering that both TTE-CO and CCABF measurements were single-time point measurements, it was impossible to time the measurements with end-expiration. Previous studies have mentioned that the reliability of the PLR test is best when using a continuous real-time measurement of CO [27]. On the contrary, other studies have quoted TTE-CO to be equivalently good compared to continuous real-time CO monitoring in assessing response to PLR testing [8]. Thirdly, this is only a single-center study and remains purely observational. Fourthly, the study was powered only to assess the correlation between Δ TTE-CO and Δ CCABF parameters with the PLR test. The sample size was not estimated based on the need for a multivariate analysis to predict factors affecting Δ TTE-CO with the PLR test. Lastly, the unavailability of dedicated ultrasound machines is a confounder when adopting this non-invasive yet highly effective tool in critical care, especially in low-resource settings [28].

## Conclusions

This study found moderate positive correlation between CO calculated using TTE-CO using LVOT VTI and CCABF at baseline. Our analysis found that the CCABF parameters were not effective in predicting a positive PLR response and using the LVOT VTI, dynamic Δ CCABF had a very poor positive correlation with Δ TTE-CO. Given the findings from our study, it may not be advisable to rely on CCABF parameters obtained during the PLR test to detect fluid responsiveness in critically ill patients.
